# Hepatitis B, Hepatitis C and HIV seroprevalence in critically ill emergency medicine department patients in a tertiary inner city hospital in Istanbul, Turkey

**DOI:** 10.12669/pjms.304.4975

**Published:** 2014

**Authors:** Tuba Cimilli Ozturk, Ozlem Guneysel, Adem Tali, Sonay Ezgi Yildirim, Ozge Ecmel Onur, Serpil Yaylaci

**Affiliations:** 1 Tuba Cimilli Ozturk, Fatih Sultan Mehmet Education and Research Hospital, Emergency Medicine Department, Istanbul, Turkey.; 2 Ozlem Guneysel, Umraniye Education and Research Hospital, Emergency Medicine Department, Istanbul, Turkey.; 3 Adem Tali, Umraniye Education and Research Hospital, Emergency Medicine Department, Istanbul, Turkey.; 4 Sonay Ezgi Yildirim, Umraniye Education and Research Hospital, Emergency Medicine Department, Istanbul, Turkey.; 5 Ozge Ecmel Onur, Fatih Sultan Mehmet Education and Research Hospital, Emergency Medicine Department, Istanbul, Turkey.; 6 Serpil Yaylaci, Acibadem University School of Medicine, Emergency Medicine Department, Istanbul, Turkey.

**Keywords:** Critically ill, Emergency department, Hepatitis B, Hepatitis C, HIV

## Abstract

***Objective:*** Emergency medicine staff is working at risk of blood-borne infections during their daily practice every time. The risk of transmission is higher when dealing with critically ill patients. Our objective was to find out the prevalence of Hepatitis B, Hepatitis C, and HIV, in critically ill red-coded emergency department patients.

***Methods:*** The study was carried out as prospective observational study between 1 September 2012 and 31 January 2013 in a tertiary inner city hospital emergency department in Istanbul, Turkey. Red triage coded patients managed in resuscitation room were enrolled.

***Results:*** One thousand patients were included during the study period. Fifty of them were HBV positive. Eighteen patients were HCV positive and 2 had both HBV and HCV. HIV was not recorded. Forty one of them were trauma patients. There were 226 unconscious or uncooperative patients. Prior blood transfusion history was present in 92 of the patients and among them 11 had HBV and 3 had HCV. Four patients or their relatives were aware of their HCV positivity. HBV positivity was already known by the patients or their relatives. Total HBV vaccination ratio was 7.4%.

***Conclusion:*** Prevalence of HCV (1.8%) and HBV(5%) seroprevalence in our study group was very low which correlated with the recent literature regarding the Turkish population. HIV was not detected during the study period. This may also be accepted as consistent with the very low number of reported cases in Turkey.

## INTRODUCTION

Health care workers (HCW) especially the emergency medicine staff are working at risk of blood-borne infections during their daily practice. The transmission of blood-borne pathogens may occur through percutaneous and mucocutaneous (i.e. contact with intact or non-intact skin, and contact with mucous membranes) routes, and sometimes, through exposure to other body fluids.^1^ Therefore it is not difficult to predict the high probability of transmission when dealing with critically ill patients in resuscitation room. It is generally difficult to obtain a true medical history, and multiple invasive procedures had to be applied on this group of patients.^2^^-^^4^

Three pathogens account for most cases of HCW exposures due to their prevalence in patients and the severity of infections they cause: hepatitis B virus (HBV), hepatitis C virus (HCV) and human immunodeficiency virus (HIV). Percutaneous injuries (80.6-87%) are the primary risk factor for HCW. The second most frequent route of exposure is projections (13-16.7%). In about 2.5-2.7% of the exposures the route of transmission couldn’t be defined.^5^

According to WHO 37% of the hepatitis B among health workers was the result of occupational exposure, while less than 10% of the HIV among health care workers is the result of a needle stick injury at work.^6^ On the other hand, previous studies have proved that working in the emergency department to be specified as a risk factor for occupational exposures.^7^^,^^8^

There are a number of prevalence studies about HBV, HCV and HIV among different populations, mostly the blood donors worldwide and in Turkey.^9^^,^^10^ However this may not reflect the exact number of patients and carriers. People, who are aware of their own seropositivity, probably do not refer to blood donation.^11^ To our knowledge there is no study showing the seroprevalence status of critically ill emergency department patients in Turkey. And more than a decade has passed since the reporting of the vast majority of the blood-borne pathogen prevalence researches in the emergency departments worldwide.^12^^-^^14^ During this time number of people living with HIV, HCV and HBV have increased due to better therapies. On the other hand HBV vaccination programs are more widespread and the compliance of HCW for the universal precautions has also increased.

The objective of this study was to provide updated prevalence information on hepatitis B, hepatitis C and HIV among critically ill red-coded emergency department patients in a tertiary hospital in Istanbul, Turkey.

## METHODS

Istanbul is the largest city in Turkey, constituting the country's economic, cultural, and historical centre. With a population of 13.9 million, it is the second-largest city after Moscow in the Europe by population within city limits. According to Istanbul City Health Directorate reports emergency department visits are about 9-10 million yearly. Umraniye Education and Research Hospital is a tertiary inner city hospital which is located next to a busy highway, with a total patient visit of 5500-6000 daily. Emergency department visits ranges between 950-1450 daily, and approximately 10-15% of them are triaged as red-coded critically ill group.

***Study Design: ***The study was carried out as a prospective observational study between 1st September 2012 and 31 January 2013 after approval of the institutional review board of the hospital. All the red triage coded paediatric patients with trauma and adult patients with or without trauma admitted to resuscitation room were enrolled during the study period. In our institution this group of patients is routinely screened for HBV, HCV and HIV. Non-traumatic paediatric patients had been evaluated in a separate area, therefore not included in the study. We included 1000 patients during the study period. Besides the demographical data, prior history of blood transfusion, homosexuality, intravenous (iv) drug abuse, the awareness of being carriers of the disease, and the HBV immunization status were assessed. Number of total invasive procedures was also noted. 

The serum samples were analyzed for hepatitis B surface antigen (HBs Ag), antibody to hepatitis C (Anti HCV) and HIV antibody (Anti HIV) by macro-ELIZA technique (Advia Centaur, Ortho-Clinical Diagnostics, Tarrytown, NY, USA) 

The data were analyzed by using SPSS 17.0 (SPSS Inc, Chicago, IL, USA). For the examination of descriptive statistical methods (frequency, percentage, mean, and standard deviation), and normal distribution, Kolmogorov-Smirnov distribution test was used. Comparisons of qualitative data were done with Pearson’s Chi-square test and Fisher’s Exact test. The results were evaluated at 95% confidence interval and the level of significance was p<0.05. The advanced level of significances were p<0.01 and p<0.001.

## RESULTS

We included one thousand patients during the study period. The number of female and male patients was equal coincidentally. Mean age was 51.7 + 23.2 years. There were 50 (5%) HBV positive and 18 (1.8%) HCV positive patients. Only 2 patients had both HBV and HCV. HIV was not recorded during the study period. Comparison of HBV positivity ratio with sex and age were found statistically insignificant. The p values were calculated as 0.147 and 0.406 respectively. Statistical comparisons of HCV positivity and age and gender were also insignificant (p =0.594 and p=0.227 respectively) (Table-I).

There were 41 (4.1%) trauma patients and 39 (3.9%) of them had external injury. Two of the trauma patients with external injury were seropositive. In terms of having trauma HBV and HCV positivity were not significant (Table-II). Two hundred and twenty six (22.6%) of the patients were unconscious or uncooperative. None of them were homosexual as they expressed. Prior blood transfusion history was present in 92 (9.2%) of the patients and among them 11 (1.1%) had HBV and 3 (0.3%) had HCV. Comparison of HBV and HCV positivity and prior transfusion showed a significant relationship (p=0,000, p=0,006) (Table-III). Intravenous drug abuse incidence was 0.6% (n=6). One of the drug abusers was found to have both HBV and HCV, and two had only HBV and one had only HCV. Two of the drug abusers were seronegative. 

There were 4 (0.4%) patients or their relatives who were aware of their HCV positivity. HBV positivity was known by 18 (1.8%) of the patients or their relatives. Total HBV vaccination ratio was 7.4%. The number of invasive procedures performed in the patients during the study period was 4620. Invasive procedures lasted for minute to maximum of seventeen miunutes. Table-IV shows the list of procedures performed.

## DISCUSSION

The recent data for HBV and HCV was released by the Turkish Association for the Study of the Liver (TASL) in 2010. Five thousand four hundred and seventy one adults from different regions of Turkey were included in the study. The general prevalence of HBV (HBS Ag +) was 4%, and HCV (Anti-HCV +) was 0.95%. Both HBV and HCV prevalence showed positive correlation with age.^15^ The mean age in our study population was 51.7 and we found HBV prevalence as 5% and HCV prevalence as 1.8%. Therefore our results seem to correlate with the report according to age groups. On the other hand there is no information about HIV prevalence in the TASL’s study. 

One of the remarkable features of the TASL report is that the HBs Ag was found positive in 2.7% of the adults between 18-29 years. This is probably because of national HBV immunization programme for new-born’s established in 1991 in Turkey. Anti HBs positivity was found 32% in the report. Total known HBV vaccination ratio was 7.4% in our study. This information was collected from the expressions of the patients and their relatives. 

 Centers for Disease Control and Prevention (CDC) estimates that approximately 50,000 people in the United States are newly infected with HIV each year.^16^ Turkey seems to be luckier than many other countries in the prevalence of AIDS. Hacettepe University HIV-AIDS Treatment and Research Centre published total number of reported HIV + patients as 5137 and AIDS patients as 1051 between 1985 and 2012 in Turkey.^17^ We did not detect any HIV and AIDS patients during the study period in our hospital. This is probably because of the very low incidence and prevalence rates of HIV and AIDS in Turkey.

Prior blood transfusion history was present in 9.2% (n=92) of the patients and among them 11 had HBV and 3 had HCV. Comparison of HBV and HCV positivity and prior transfusion showed a significant relationship as expected. Homosexuality may be denied particularly by the patients and relatives, because of not being disclosed. Intravenous drug abuse was reported by only 6 patients. There were 4 patients or their relatives who were aware of their HCV positivity. HBV positivity was known by 18 of the patients or their relatives. As we stated before these results may be unreliable in this special population due to unpredictable medical past histories.

HCW especially the emergency medicine staff is working at risk of blood-borne infections during their daily practice. According to the reports of WHO 37% of the hepatitis B among HCW was the result of occupational exposure. Infection with the hepatitis B virus is 95% preventable with immunization but less than 20% of HCW in some regions of the world have received all three doses needed for immunity. While less than 10% of the HIV among HCW is the result of an exposure at work, needle stick injuries, the cause of 95% of the HIV occupational seroconversions, are preventable with practical, low-cost measures and have the co-benefit of preventing exposure to other blood-borne viruses and bacteria.^6^ As it is stated before, the most probable transmission route is percutaneous injuries.^1^

**Table-I:** Comparison of HBV and HCV according to age and gender

		**HBV(-)**		**HBV (+)**		**p**	**HCV (-)**		**HCV (+)**		**p**
		**n**	**%**	**n**	**%**		**n**	**%**	**n**	**%**	
**Age (years)**	**<20**	101	% 10,6	4	% 8,0	X^2^=2,906 p=0,406	105	% 10,7	0	% 0,0	X^2^=4,339 p=0,227
	**21-40 **	238	% 25,1	8	% 16,0		242	% 24,6	4	% 22,2	
	**41-60 **	230	% 24,2	14	% 28,0		241	% 24,5	3	% 16,7	
	**>61**	381	% 40,1	24	% 48,0		394	% 40,1	11	% 61,1	
**Gender**	**Female **	480	% 50,5	20	% 40,0	X^2^=2,105 p=0,147	491	% 50,0	9	% 50,0	X^2^=0.000 p= 0.594
	**Male **	470	% 49,5	30	% 60,0		491	% 50,0	9	% 50,0	

**Table-II:** Comparison of HBV and HCV positivity in terms of having trauma

		**Trauma (-)**		**Trauma (+)**		**p**
		**n**	**%**	**n**	**%**	
**HBV**	**-**	910	% 95,8	40	% 4,2	X^2^=0,590 p=0,442
	**+**	49	% 98,0	1	% 2,0	
**HCV**	**-**	942	% 95,9	40	% 4,1	X^2^=0,099 p=0,753
	**+**	17	% 94,4	1	% 5,6	

**Table-III:** Comparison of HBV and HCV patients in terms of prior transfusion history

		**Prior transfusion (-)**		**Prior transfusion (+)**		**p**
		**n**	**%**	**n**	**%**	
**HBV**	**-**	872	% 91,8	78	% 8,2	X^2^=22,268 p=0,000
	+	36	% 72,0	14	% 28,0	
**HCV**	**-**	895	% 91,1	87	% 8,9	X^2^=7,573 p=0,006
	**+**	13	% 72,2	5	% 27,8	

**Table-IV:** Invasive procedures applied

**Procedures**	**Frequency (n)**	**Percentage (%)**
Periferal iv line	997	99.7
Urine catheter	443	44.3
Nasogastric catheter	319	31.9
Intubation	206	20.6
Suture	40	4
Arterial line	17	1.7
Thoracentesis-paracentesis	9	0.9
Thorax tube	8	0.8
Central iv line	7	0.7
Intraosseos line	3	0.3
Cricotirotomy	3	0.3

In 2007 the Healthcare Infection Control Practices Advisory Committee (HICPAC) updated the guideline for isolation precautions and preventing transmission of infectious agents in healthcare settings.^18^ However compliance with the universal precautions for preventing transmission of blood-borne infections was found to be inadequate in different studies.^19^-^21^ The invasive procedures on our study population lasted for one minute to seventeen minutes. Total number was 4620. Unfortunately we did not record the total number of interventional trials. Because of the clinical instability of the study population, it was generally troublesome even to put an intravenous peripheral access. Therefore multiple trials had to be done by the staff. Considering the very rapid evaluation of the red coded critically ill patients in the resuscitation room the risk of accidents is probably higher than other working areas. The frequency of invasive procedures in our study demonstrated the risk status of the study environment. Unfortunately according to our clinical observation, HCW working in the resuscitation area did not routinely apply the standard universal precautions adequately despite this high frequency.

Although training and education remain key preventive measures for improving HCW compliance with improved standard precautions, safety-engineered devices (SEDs) (retractable syringes, needle-free intravenous systems, winged butterfly needles) are now being increasingly promoted for use in decreasing the rate of percutaneous injuries.^22^^,^^23^

***Limitations:*** The results about blood transfusion history, homosexuality, immunization status, Intravenous drug abuse ratios in our study probably did not reflect the true picture due to the special nature of our study population. Standard screening tests may not catch the viruses in incubation period. We did not search the Anti HBs in the study. Therefore this may not reflect the exact immunization status either. In addition, 22.6% (n=226) of patients being unconscious or uncooperative in our study group shouldn’t be ignored. 

## CONCLUSION

Prevalence of HCV (1.8%) and HBV (5%) seroprevalence in our specific study group was very low which correlated with the recent literature regarding the Turkish population. HIV was not detected during the study period. This may also be accepted as consistent with the very low number of reported cases in Turkey. However the emergency department staff must be aware of their risky work place and take necessary measures assuming that every patient might have a contagious disease. 

## Authors' Contributions:

**TCO and OG:** Designed and carried out the research, collected the data, interviewed the patients, coordinated the study and prepared the manuscript.

**AT and SEY:** Provided assistance in designing and conducting the research.

**OEO and SY:** Participated in the analysis and interpretation of the data, corrected the English language and revised further statistical data.

All authors have read and approved the content of the manuscript.

## Notes

***Disclosure: ***Authors have no conflict of interests, and the work was not supported or funded by any drug company.
